# Evaluating the Influence of Spatial Resampling for Motion Correction in Resting-State Functional MRI

**DOI:** 10.3389/fnins.2016.00591

**Published:** 2016-12-27

**Authors:** Lisha Yuan, Hongjian He, Han Zhang, Jianhui Zhong

**Affiliations:** ^1^Center for Brain Imaging Science and Technology, Key Laboratory for Biomedical Engineering of Ministry of Education, College of Biomedical Engineering and Instrumental Science, Zhejiang UniversityHangzhou, China; ^2^Center for Cognition and Brain Disorders, Hangzhou Normal UniversityHangzhou, China; ^3^Department of Radiology and BRIC, University of North Carolina at Chapel HillChapel Hill, NC, USA; ^4^Department of Imaging Sciences, University of RochesterRochester, NY, USA

**Keywords:** head motion correction, spatial resampling, motion regression approaches, resting-state functional MRI

## Abstract

Head motion is one of major concerns in current resting-state functional MRI studies. Image realignment including motion estimation and spatial resampling is often applied to achieve rigid-body motion correction. While the accurate estimation of motion parameters has been addressed in most studies, spatial resampling could also produce spurious variance, and lead to unexpected errors on the amplitude of BOLD signal. In this study, two simulation experiments were designed to characterize these variance related with spatial resampling. The fluctuation amplitude of spurious variance was first investigated using a set of simulated images with estimated motion parameters from a real dataset, and regions more likely to be affected by spatial resampling were found around the peripheral regions of the cortex. The other simulation was designed with three typical types of motion parameters to represent different extents of motion. It was found that areas with significant correlation between spurious variance and head motion scattered all over the brain and varied greatly from one motion type to another. In the last part of this study, four popular motion regression approaches were applied respectively and their performance in reducing spurious variance was compared. Among them, Friston 24 and Voxel-specific 12 model (Friston et al., 1996), were found to have the best outcomes. By separating related effects during fMRI analysis, this study provides a better understanding of the characteristics of spatial resampling and the interpretation of motion-BOLD relationship.

## Introduction

Functional magnetic resonance imaging (fMRI) is a technique that utilizes the blood oxygen level dependent (BOLD) effect to indirectly detect the neuronal activity. fMRI studies have shown explosive growth and a wide range of clinical applications (Greicius, 2008; Lee et al., 2013). Since neuronal activities can cause BOLD signal changes of a few percent at best locally in the brain, even slight head motion has been recognized as a big confounding factor due to the long scan time and low signal changes in fMRI. Head motion could add spurious signals, leading to false activation or deactivation (Aguirre et al., 1998; Miezin et al., 2000), and yielding distance-dependent motion artifacts on functional connectivity in resting state fMRI studies (Power et al., 2012; Satterthwaite et al., 2012; Van Dijk et al., 2012). Therefore, the statistical inference of fMRI results would be compromised by head motion, especially for studies in the pediatric and elderly populations, or the seriously ill, who are often more prone to move during the scan.

Many efforts have been made to minimize the influence from head motion. For example, foam pad, bite bars or facial masks have been used to fix the head position and alleviate the influence of head motion directly. However, these restraint devices can make the subject uncomfortable and distracted thus more likely to trigger undesired neuronal activities (Lueken et al., 2012). On the other side, prospective motion correction maintains a relative invariable position between the imaging volume and the moving brain by tracking head motion and continuously updating the imaging pulse sequence (Maclaren et al., 2013), but the special requirements for sequence design reduce its effectiveness in routine experiments.

Image realignment has been commonly performed to correct head motion in fMRI retrospectively (Friston et al., 1995; Zitova and Flusser, 2003). It usually consists of motion parameter estimation using a weighted least-squares cost function and spatial resampling along with an interpolation method to realize rigid-body inter-frame spatial registration within subject. There have been studies focused on the accuracy of motion estimation (Kim et al., 1999; Ardekani et al., 2001; Oakes et al., 2005), and also the reliability of resampling methods (Hajnal et al., 1995; Jenkinson et al., 2002; Bannister et al., 2004) to improve the registration performance. Simulations have been used for qualitative assessment of registration performance in some studies (Ardekani et al., 2001; Morgan et al., 2001; Oakes et al., 2005). The initial signal of task-fMRI was set with known activation locations and magnitudes using a computer-generated phantom. Motion correction tools were then applied and the alteration of initial signal was calculated. For instance, one study found that image realignment process could introduce false information in motion free data, and indicated that spatial resampling be a cause of observed errors (Morgan et al., 2001). When it is possible to predefine activation in task-fMRI, the scenario of resting-state is quite different. The functional signal in resting-state is spontaneous and has no prior knowledge of its temporal change. The spontaneous fluctuation has relatively small amplitude and is more easily contaminated by physiological confounding as well as other noise, such as motion artifacts. Lastly, a typical resting-state fMRI study could be interested in large-scale networks or even the entire brain. This is distinct from the task-fMRI, where only specific regions are usually activated. All these facts bring in our first motivation, considering that the influence of image realignment could be dissimilar and more critical in resting state to some extent. As a second motivation, it is also our interest to evaluate the widely used motion regression strategies. Currently, regression of the six realignment estimates and their expansions have been considered as a common practice to suppress motion-related variance. Although there have been test-retest studies regarding this issue, their outcomes have not evaluated with a simulation metric yet, where the ground truth can be defined properly.

In this paper, simulation experiments were designed to characterize spurious variance (SV) caused by volumetric registration. The fluctuation amplitude of SV was first investigated using a set of simulated images with estimated motion parameters extracted from a real dataset. The motion-SV relationship was then investigated using simulated data with three typical types of motion with defined parameters. In addition, four common motion regression approaches based on realignment estimates were adopted as nuisance regressors respectively, and their effects on the reduction of SV were compared.

## Methods

### Data acquisition

The MR experiment in this study was approved by the research ethics review board of Hangzhou Normal University. Sixty-five healthy adults, who were right-handed and had no history of neurological or psychiatric illness, were enrolled in this study, and signed the informed consent. All subjects were instructed to stay still with eyes closed, as they were scanned on a 3.0T GE MR750 system (GE healthcare, Waukesha, WI). Firstly, T1-wighted structural images were acquired using a 3D sagittal FSPGR sequence with the following parameters: FOV = 250 × 250 × 180 mm^3^, matrix size = 250 × 250 × 180, TR/TE/FA = 8100 ms/3.1 ms/8°, TI = 450 ms, bandwidth = 31.25 kHz, and the total scan time = 5 min. Then functional images were acquired using an axial GRE-EPI sequence with the following parameters: FOV = 220 × 220 mm^2^, matrix size = 64 × 64, TR/TE/FA = 2000 ms/30 ms/90°, 3.2 mm slice thickness, 43 slices, time points = 240, parallel acceleration = 2, and the total scan time = 8 min.

Image pre-processing was performed using the DPARSFA toolbox (http://www.restfmri.net) (Yan and Zang, 2010) and SPM8 software (http://www.fil.ion.ucl.ac.uk/spm). For functional images of every subject, the first five volumes were discarded for reaching T1 equilibrium. Slice timing was corrected for the time shifts among different acquisitions within each volume. Each volume was then realigned to the first volume to estimate motion parameters and correct for head motion, and two orders of polynomial trends were regressed out as basic nuisance covariates; these two steps were also held fixed for all following simulation experiments once simulated data were created. Data of 44 subjects (gender: Female (14), Male (30); age: 22.8 ± 2.5 years) with the motion threshold of 1.5 mm or 1.5 degree and registration outcomes of less distortion were selected for the subsequent simulations.

### Simulation experiments

A series of simulated fMRI data were created to evaluate the influence of spatial resampling. For each subject, the rigid-body motion parameters were extracted from functional images and modified to mimic the realistic motion. During the simulation, these motion parameters were applied to the first functional image (reference image), and the spatial resampling using the 4th B-spline interpolation was adopted to generate motion-contaminated simulated images. Next, an estimation of motion correction was performed to calculate the motion parameters of the simulated data, and then the same resampling method was used to achieve image realignment. Therefore, the voxel-wise fluctuation in time series among motion-corrected simulated images could be characterized as spurious variance (SV), which was mainly affected by spatial resampling. In this study, the fluctuation amplitude of SV, as well as the correlation relationship between SV and head motion, was respectively investigated by the following two simulation experiments.

#### Simulation 1

In order to realistically simulate the temporal characteristics of subject movement, the motion parameters estimated from each subject were used in this simulation experiment. Each simulated dataset was created by resampling the first functional image of each subject with the estimated motion parameters.

Instead of amplitude of low frequency fluctuation (ALFF) in frequency domain (Zang et al., 2007), the standard deviation (SD) was chosen as a metric to measure the fluctuation amplitude of each voxel in time domain. The SD map of each simulated dataset in Simulation 1 was calculated in native space to evaluate the fluctuation amplitude of SV. Each SD map was mean-centered and variance-normalized within the brain (so called the z-standardization). All of the z-standardized SD maps were then converted to the Montreal Neurological Institute (MNI) atlas space by spatial normalization with T1 image unified segmentation, which followed by smoothing with a Gaussian kernel of 6 mm full-width half-maximum (FWHM). Finally, one sample *t*-test was applied to find affected regions with relatively larger amplitude of SV in group.

The SV introduced by spatial resampling in Simulation 1 was at comparable level with that from human studies due to comparable motion levels. Therefore, the voxel-wise relative amplitude of SV to BOLD signal was evaluated from the ratio map between SD map of each dataset in Simulation 1 and that of the corresponding dataset in human data.

(1) $$\text{Ratio map }=\;\frac{\text{SD }(\text{spurious variance})}{\text{SD }(\text{BOLD signal})},$$

The individual ratio map was also z-standardized, normalized and smoothed. One sample *t*-test was then applied on all ratio maps to find affected regions with a relatively larger ratio (the ratio of SV and BOLD signal compared with the mean ratio within individual's brain) in-group.

Furthermore, the magnitude of the influence of spatial resampling was also evaluated. Firstly, taking these affected regions as a mask and recalculating ratio maps without the z-standardization, the exact ratio averaged within the mask in MNI space was acquired from each simulated dataset. Secondly, as a concurrent report of statistical hypothesis testing, the effect size *Cohen's d* measure was also calculated to provide the magnitude of resampling effects.

**Figure 1:**
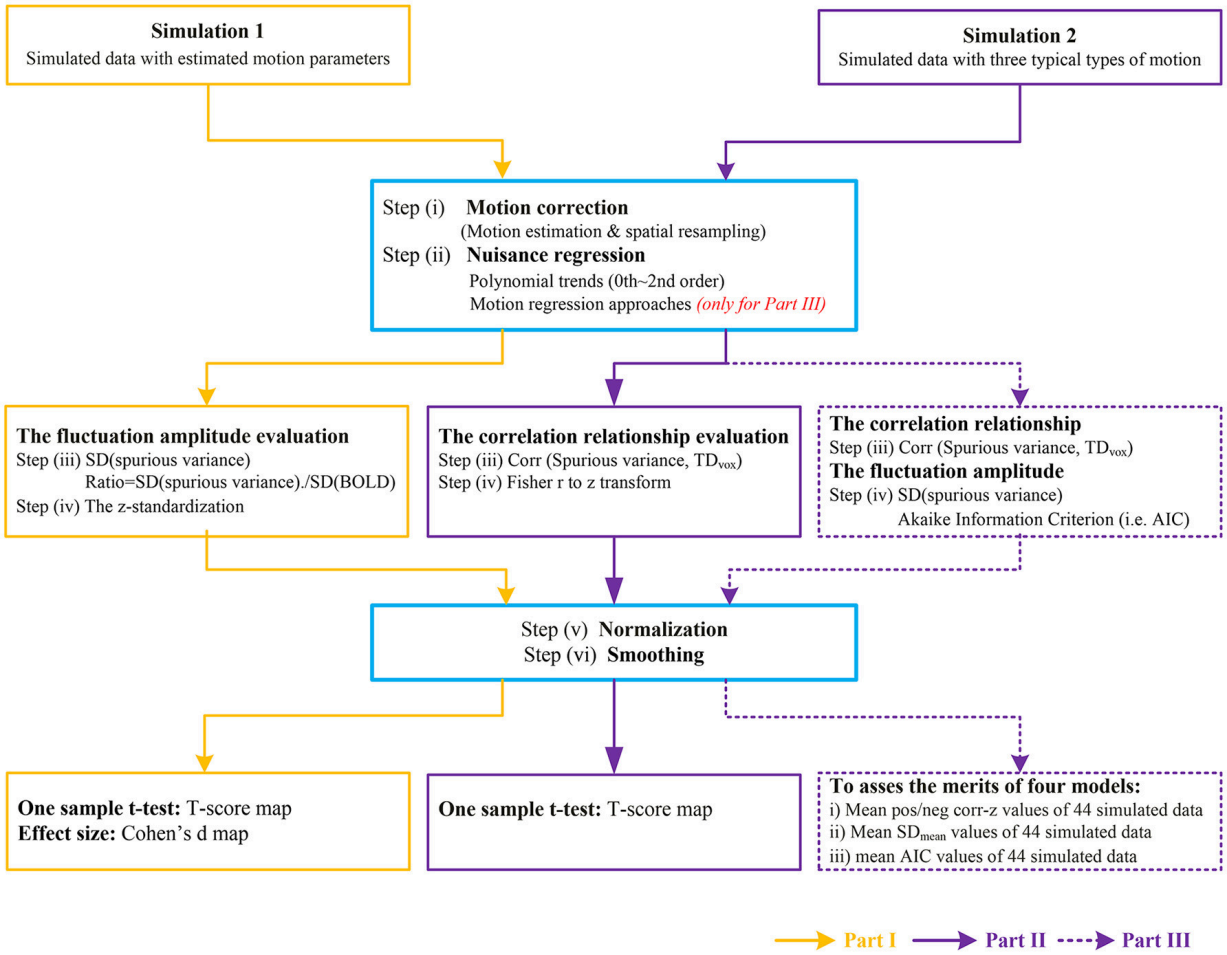
**The flow chart of image processing for simulated data**. Part I represents the data processing to investigate the fluctuation amplitude of SV with Simulation 1. Part II represents the data processing to investigate the correlation relationship between SV and head motion with Simulation 2. The purple dash arrows depict the performance evaluation of four motion regression approaches as implemented in Part III, in which the abilities of reducing the correlation between SV and head motion or decreasing the amplitude of SV were evaluated in different types of head motion.

A brief schematic of image processing pipeline for simulation 1 was illustrated in Part I of Figure 1.

#### Simulation 2

**Figure 2:**
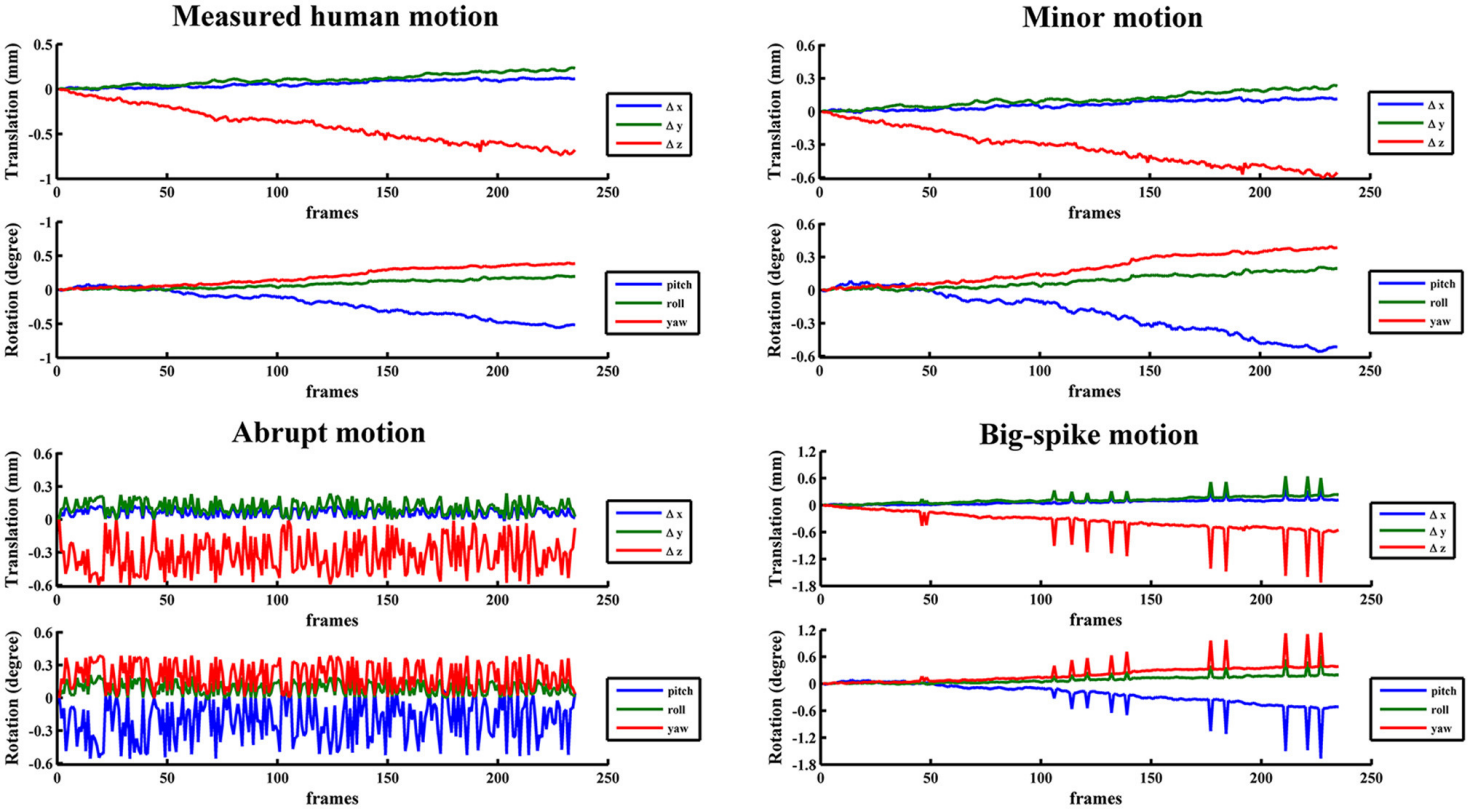
**The estimated motion from a representative subject and three corresponding typical types of motion were illustrated with head motion curves**.

In view of the individual and population difference, the timing diversity of subject movement should not be neglected (Power et al., 2012; Christodoulou et al., 2013). Some subjects may move slowly in a small position range (minor motion), some oscillate continuously and quickly at displacements below the certain threshold (abrupt motion), while others move slowly but sometimes suddenly have a burst of movement beyond the threshold value (big-spike motion). As most scenarios of head motion could be categorized to one of the three motion types or their combinations, three typical types of motion were simulated based on estimated human motion parameters: (1) *minor motion*, of which the estimated motion parameters were scaled in the range of −0.6~0.6 mm or −0.6~0.6 degree (Murphy et al., 2013), by multiplying the estimated motion parameters with the ratio of 0.6 and the absolute maximum value in a certain direction if the absolute maximum value in any direction exceeded 0.6 mm or 0.6 degree; (2) *abrupt motion*, of which the frames of minor motion were randomly permuted; (3) *big-spike motion*, of which the minor motion in randomly selected 5% frames were tripled. These three types of motion from a representative subject were illustrated with head motion curves in Figure 2. Three groups of simulated motion-contaminated images were respectively created by applying one type of head motion parameters to the first functional image of each subject.

The temporal pattern of SV was then observed in Simulation 2. Considering the regional heterogeneity of head motion and assuming no cumulative temporal motion effects in simulated images, the voxel-wise total displacement (TD_vox_) at each time point was utilized to measure head motion (Satterthwaite et al., 2013; Yan et al., 2013),

(2) $${\text{TD}}_{\text{vox},\text{t}}=\sqrt{{\left({\text{x}}_{\text{t}}-{\text{x}}_{\text{t }=\;0}\right)}^{2}+{\left({\text{y}}_{\text{t}}-{\text{y}}_{\text{t }=\;0}\right)}^{2}+{\left({\text{z}}_{\text{t}}-{\text{z}}_{\text{t }=\;0}\right)}^{2}},$$

where (x_t_, y_t_, z_t_) represented the coordinate of a voxel at time t, and (x_0_, y_0_, z_0_) represented the initial coordinate of the same voxel, which was defined as the product of voxel-to-world mapping affine matrix and the voxel index in the image space. The coordinate of the voxel at any time (x_t_, y_t_, z_t_), was derived according to the initial coordinate and the rigid-body transformation matrix between the current time point and the initial time point. The SV-TD_vox_ correlation maps were calculated for each type of motion, and then transformed to *z*-value maps through Fisher's r-to-z transformation. After normalization and smoothing, the significantly correlated regions in-group were revealed by one sample *t*-test at a corrected per-voxel threshold of *p* = 0.01 in the MNI space. A brief schematic of image processing pipeline for simulation 2 was illustrated in Part II of Figure 1.

### Motion regression approaches

Generally motion-related artifacts were reduced by retrospective motion regression approaches. In this study, four regression approaches based on realignment estimates were used as additional nuisance covariates to decrease the influence of spurious variance. These approaches are respectively, (1) Rigid-body 6-parameter model (Satterthwaite et al., 2013; Yan et al., 2013); (2) Derivative 12-parameter model (Andrews-Hanna et al., 2007; Power et al., 2012); (3) Friston 24-parameter model (Friston et al., 1996; Power et al., 2014); and (4) Voxel-specific 12-parameter models (Friston et al., 1996; Yan et al., 2013). All types of motion in Simulation 2 were utilized to evaluate the effectiveness of four approaches from two aspects, (1) the reduced correlation coefficient between SV and head motion; (2) the decreased amplitude of SV.

For each type of motion, the SV-TD_vox_ correlation maps were calculated after motion regression with each model. After Fisher's r-to-z transformation, normalization and smoothing, the mean positive / negative correlation *z*-value was extracted from each simulated dataset. The less residual relevance between SV and head motion remained, the better model it was. To assess the merits of four models, one-way ANOVA and *post-hoc* multiple comparison tests were performed on the mean positive / negative correlation *z* values of 44 simulated data across four approaches.

At the same time, for each type of motion, the SD map of each simulated dataset was calculated after basic nuisance covariates, as well as after each model regression. Each SD map was then normalized and smoothed, and the mean of SD values within the brain (SD_mean_) was calculated. Smaller residual signal amplitude from the better model would also result in the smaller SD_mean_ over the brain. And then one-way ANOVA and *post-hoc* multiple comparison tests were performed on SD_mean_ values of 44 simulated data across four approaches. As higher-order regression approaches may fit the influence of spatial resampling better than lower-order ones, information criterion was taken to evaluate the efficacy of different model-order approaches. In this work, Akaike information criterion (AIC) was used to balance model fit and model complexity (Akaike, 1974). The average of AIC values within the brain was calculated at an individual level, and then the mean AIC values of each model were obtained across simulated data. A lower AIC value means a more successful model, and model superiority can be concluded if the delta-AIC value is >2.

The image processing pipeline for the performance evaluation of motion regression approaches was marked with purple dash arrows as Part III in Figure 1.

## Results

### The characteristics of SV

**Figure 3:**
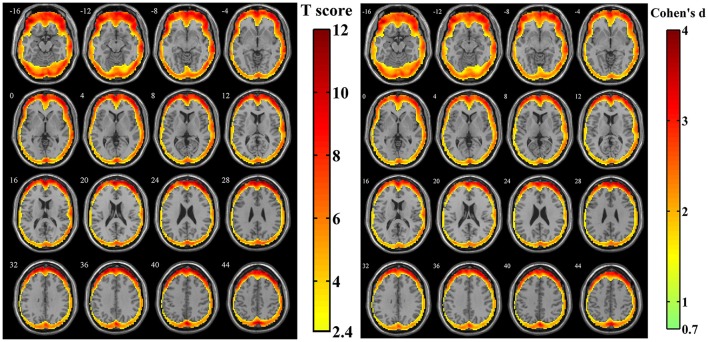
**The t-score map and *Cohen's d* map obtained from 44 z-standardized SD maps in Simulation 1**. Yellow-red areas illustrated affected regions which had a relatively larger amplitude of SV in individual level with higher possibilities (left image: *n* = 44, per-voxel threshold of *p* = 0.01 and a FWE-corrected threshold of *p* = 0.05 via the bug-fixed 3dClustSim, *t* > 2.416) or strong effect size (right image: *n* = 44, *Cohen's d* > 0.7369).

For the simulated data with estimated human motion parameters in Simulation 1, spatial distribution of the amplitudes of SV was investigated from the z-standardized SD maps. The result of one sample *t*-test revealed that the regions more likely to be affected by volumetric registration appeared around the peripheral regions of the cortex, including most areas of the frontal cortex, the middle, and superior temporal gyrus, middle occipital gyrus and a tiny area of the inferior parietal lobule (Figure 3, *n* = 44, per-voxel threshold of *p* = 0.01 and a FWE-corrected threshold of *p* = 0.05 via the bug-fixed 3dClustSim, *t* >2.416). Besides, the magnitudes of *Cohen's d* within these regions were >0.7369, indicating consistently higher amplitudes at an individual level.

**Figure 4:**
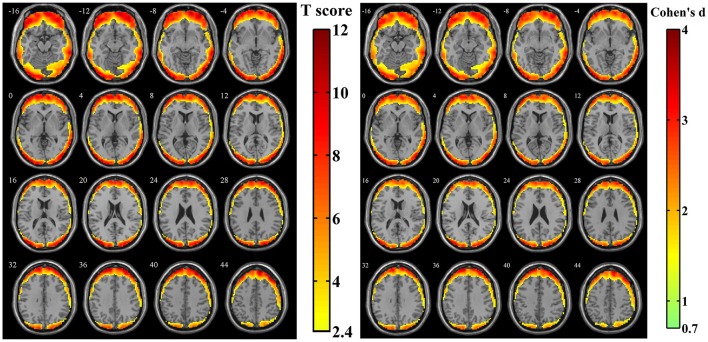
**The t-score map and *Cohen's d* map obtained from 44 z-standardized ratio maps (SD maps in Simulation 1 divided by SD maps in human data)**. Yellow-red areas illustrated affected regions which had a relatively larger ratio of SV and BOLD signal in individual level with higher possibilities (left image: *n* = 44, per-voxel threshold of *p* = 0.01 and a FWE-corrected threshold of *p* = 0.05 via bug-fixed 3dClustSim, *t* > 2.416) or strong effect size (right image: *n* = 44, *Cohen's d* > 0.7369).

After calculating the z-standardized ratio maps, the relative amplitude of SV to BOLD signal was investigated and the relatively significantly affected area was showed in Figure 4 (*n* = 44, per-voxel threshold of *p* = 0.01 and a FWE-corrected threshold of *p* = 0.05 via bug-fixed 3dClustSim, *t* > 2.416). We found that spatial distribution of these regions in Figure 4 was almost overlapped with that in Figure 3, except the extended areas in the inferior temporal gyrus as well as the absent areas in the medial frontal gyrus and superior temporal gyrus. Examination of the effect size (using *Cohen's d*) further supported higher relative amplitude of SV to BOLD signal in these regions. Moreover, the exact ratios within these regions were (13.07 ± 6.00)% across subjects.

**Figure 5:**
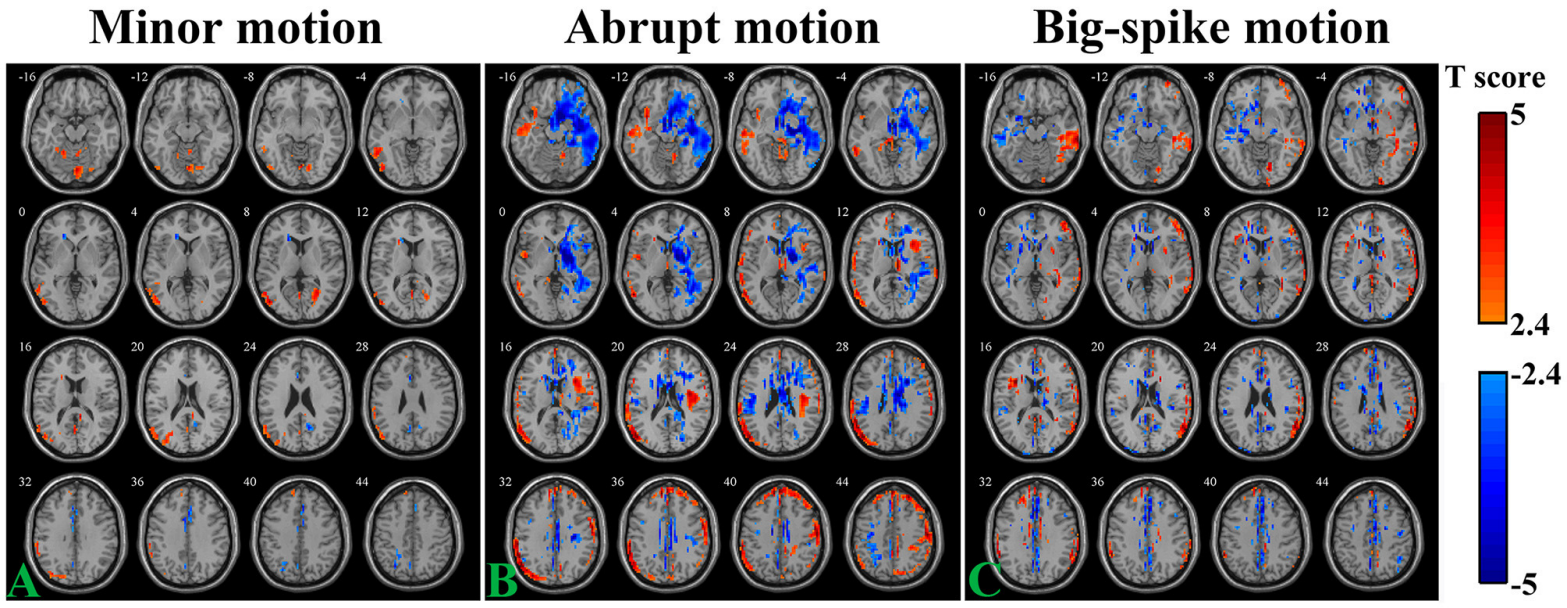
**Voxel-wise correlation analysis between SV and TD_vox_ in three motion types** (**A**, minor motion; **B**, abrupt motion; **C**, big-spike motion) of Simulation 2. Affected regions showing significantly negative / positive correlation (*n* = 44, per-voxel threshold of *p* = 0.01 and a FWE-corrected threshold of *p* = 0.05 via bug-fixed 3dClustSim, |*t*| > 2.416) were characterized in light blue to dark blue and light red to dark red, respectively.

The SV-TD_vox_ correlation relationship was explored in different motion types of Simulation 2. As illustrates in Figure 5, areas where SV significantly correlated with TD_vox_ not only spread around the peripheral regions of the cortex, but scattered over the entire brain (*n* = 44, per-voxel threshold of *p* = 0.01 and a FWE-corrected threshold of *p* = 0.05 via bug-fixed 3dClustSim, |t|>2.416). Regarding the minor motion type, negative correlation was exhibited in the cingulate gyrus, parietal lobe, and near the CSF, while positive correlation was mainly exhibited in the middle/superior temporal, occipital gyrus, and inferior parietal gyrus (Figure 5A). For the abrupt motion type, the negative correlation in the temporal gyrus and frontal gyrus were prominent, while positive correlation were mainly located in the frontal lobe and parietal lobe (Figure 5B). The result of simulated data with big-spike motion showed that negative correlation distributed in the middle frontal lobe, temporal lobe and cingulate gyrus, and that positive correlation distributed in the temporal lobe, inferior parietal lobe and superior frontal gyrus (Figure 5C).

### The merits of motion regression approaches in reducing SV

**Figure 6:**
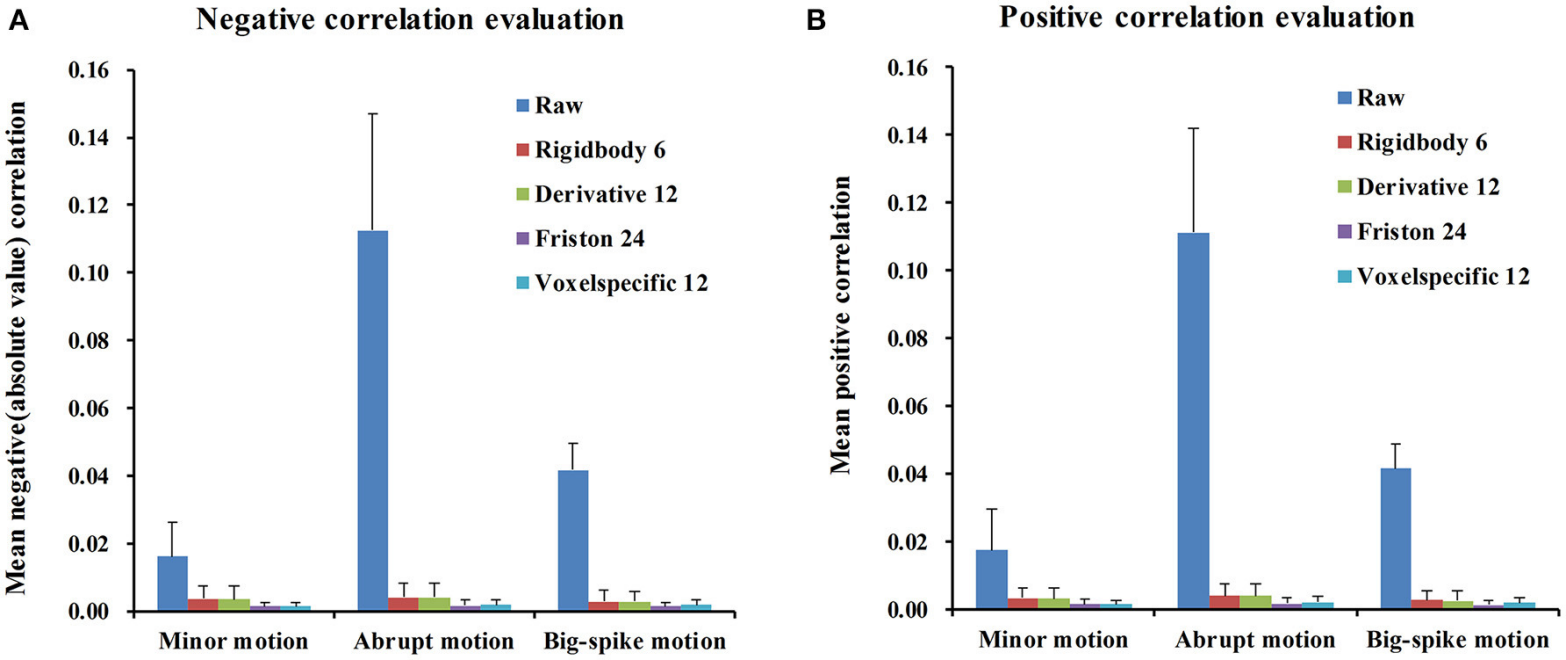
**The performance of four regression approaches in reducing the correlation *z*-values between SV and TD_vox_**. All of four regression approaches showed some ability to reduce the relevance of SV and TD_vox._ **(A)** The result of negative correlation evaluation indicated that Friston 24 and Voxel-specific12 had a better performance than other models for minor motion type and abrupt motion type. Nevertheless, there was no significantly different performance among all of four models for big-spike motion. **(B)** The result of positive correlation evaluation was basically consistent with that of negative correlation, except that Friston 24 model was better than Rigidbody 6 model in reducing positive correlations for big-spike motion. “Raw” showed the mean positive / negative correlation *z*-values of 44 simulated data after regular processing without motion regression.

For each type of motion, the average and standard deviation of mean negative and mean positive correlation *z*-values from 44 simulated data were calculated and summarized in Table 1. All of four regression approaches showed some ability to reduce the relevance of SV and TD_vox_ (Figure 6). The result of one-way ANOVA followed by multiple comparison of Bonferroni's correction across regression approaches demonstrated that Friston 24 and Voxel-specific 12 models had a better performance in reducing negative and positive correlation relationships for both minor and abrupt motion types. For big-spike motion type, there was no significant difference among four approaches in reducing negative correlations, but Friston 24 model was slightly better in reducing positive correlations than Rigidbody 6. (Please view Tables S1–S3 in Supplementary material documents for more details about statistical parameters)

**Table 1:** **The average and standard deviation of mean negative and mean positive correlation z values from 44 simulated data were summarized**.

		**Minor motion**		**Abrupt motion**		**Big-spike motion**	
		**Negative**	**Positive**	**Negative**	**Positive**	**Negative**	**Positive**
Raw	mean	−0.0161	0.0175	−0.1125	0.1112	−0.0418	0.0415
	std	0.0101	0.0119	0.0347	0.0309	0.0077	0.0075
Rigidbody 6	mean	−0.0037	0.0033	−0.0042	0.0039	−0.0029	0.0027
	std	0.0040	0.0031	0.0042	0.0036	0.0032	0.0028
Derivative 12	mean	−0.0035	0.0032	−0.0042	0.0039	−0.0028	0.0026
	std	0.0039	0.0030	0.0041	0.0035	0.0031	0.0028
Friston 24	mean	−0.0014	0.0015	−0.0018	0.0018	−0.0015	0.0014
	std	0.0013	0.0015	0.0016	0.0018	0.0012	0.0011
Voxel-specific 12	mean	−0.0015	0.0016	−0.0020	0.0022	−0.0020	0.0019
	std	0.0011	0.0012	0.0015	0.0018	0.0016	0.0014

“*Raw” showed the mean positive / negative correlation z-values of 44 simulated data from regular processing without motion regression*.

**Figure 7:**
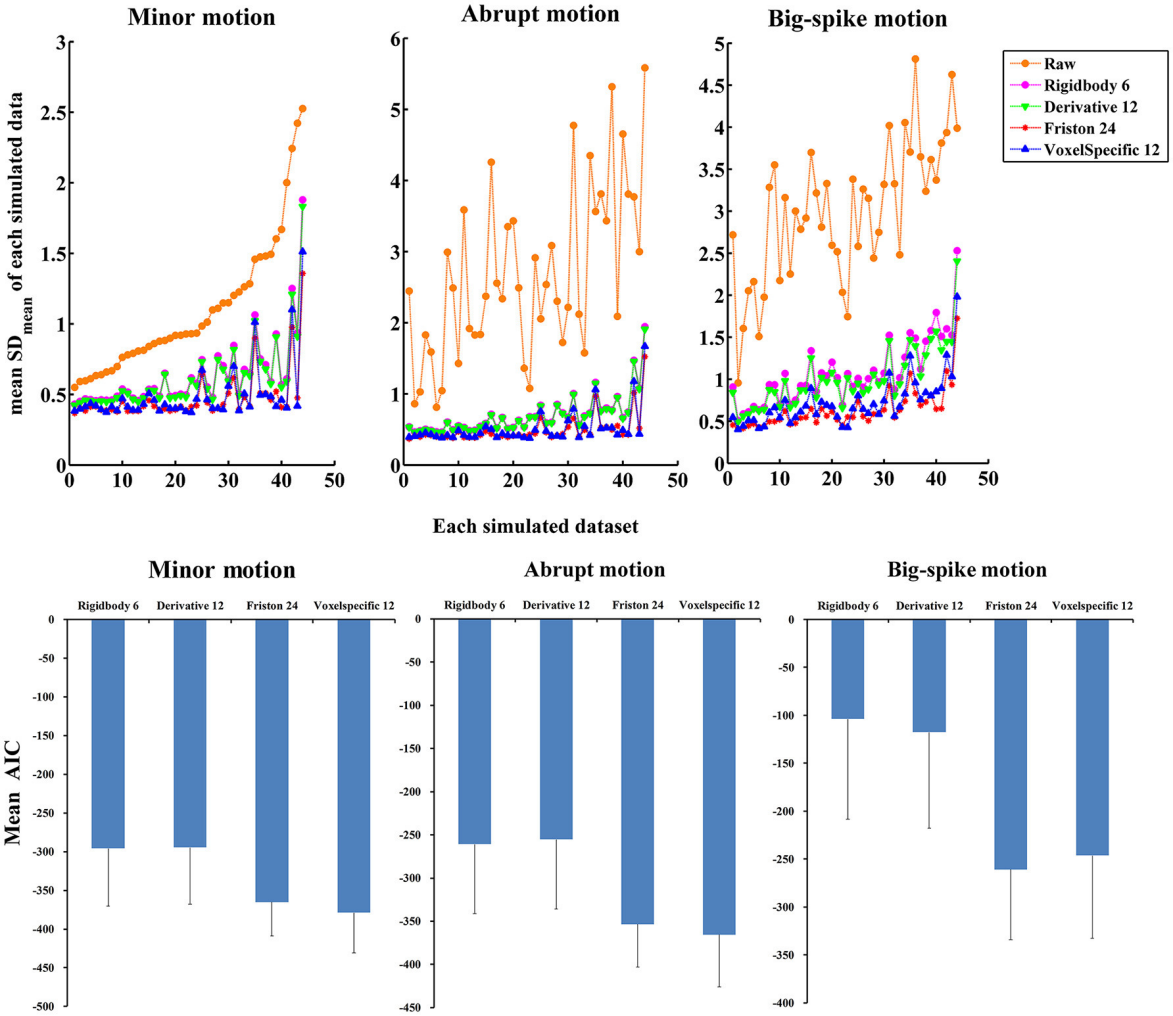
**The performance of four approaches in reducing the fluctuation amplitudes of SV**. (Top) SD_mean_ value of each simulated dataset was plotted, and all regression approaches reduced a certain degree of the fluctuation amplitudes of SV. Among them, Friston 24 and Voxel-specific12 models had a better performance than other models for all types of motion. (Bottom) Mean AIC values across simulated data were plotted for each regression approach, and Friston 24 and Voxel-specific12 models showed lower AIC values for all types of motion. The result of delta-AIC would recommend Voxel-specific 12 model for minor motion and abrupt motion, while Friston 24 model was the priority for big-spike motion. The Rigidbody 6 and Derivative 12 models were deprecated from further considerations.

In addition, the average and standard deviation of SD_mean_ values from 44 simulated data were also calculated for each motion type, as summarized in Table 2. All regression approaches reduced the fluctuation amplitudes of SV in some degree (Figure 7). The result of one-way ANOVA followed by multiple comparison of Bonferroni's correction indicated that Friston 24 and Voxel-specific 12 models had a better performance for all types of motion, but that there was no significant difference between these two approaches. (Please view Table S4 in Supplementary material document for more details about statistical parameters) Moreover, the evaluation of mean model AIC showed a similar conclusion about the performance of regression approaches, as better fitness of higher-order approaches had lower mean AIC values. The result of delta-AIC would recommend Voxel-specific 12 model for minor motion and abrupt motion, while Friston 24 model was the priority for big-spike motion. The Rigidbody 6 and Derivative 12 models were deprecated from further considerations.

**Table 2:** **The average and standard deviation of SD_mean_ values from 44 simulated data were summarized**.

		**Minor motion**	**Abrupt motion**	**Big-spike motion**
Raw	mean	1.1014	2.6730	2.9909
	std	0.4840	1.2029	0.8312
Rigidbody 6	mean	0.6330	0.7007	1.0833
	std	0.2620	0.2834	0.3896
Derivative 12	mean	0.6160	0.6911	1.0086
	std	0.2534	0.2787	0.3577
Friston 24	mean	0.4742	0.4933	0.6343
	std	0.1831	0.2073	0.2358
Voxel-specific 12	mean	0.4913	0.5147	0.7199
	std	0.2158	0.2426	0.2870

“*Raw” showed the mean of SD_mean_ values of 44 simulated data from regular processing without motion regression*.

The above evaluation methods suggest that Friston 24 and Voxel-specific 12 models have a better ability to effectively reduce, but not completely eliminate SV.

## Discussion

This study aimed to evaluate the influence of spatial resampling and the merits of motion regression approaches with a simulation metric. We hence simulated different types of head motion, and evaluated the effect of spatial resampling as well as the efficacy of regression approaches with respect to the signal variance and signal-motion relationship.

In this study, motion parameters were applied on a single reference image to generate a series of images containing head motion, and the motion-contaminated images were corrected during motion correction. It should be noted that any estimation algorithm could not be perfectly accurate and there usually exists deviation between the given and estimated motion parameters. In all simulated data of Simulation 1, the max estimation errors in each direction were <0.0381 mm (x), 0.0738 mm (y), 0.0330 mm (z), 0.0690° (α), 0.0802° (β), Z and 0.0396° (γ) respectively. Compared with the voxel size (3.44 × 3.44 × 3.2 mm^3^), the estimation errors were limited. While it is crucial to pursue a perfect estimation, our study used these estimated parameters in further simulation and comparison in order to follow a common procedure that would occur in an fMRI study. Therefore, the variation of intensity between image frames was mainly affected by spatial resampling twice, first during generation of simulated motion data and second during the motion correction. As far as we know, little attention has been paid to the influence of spatial resampling for resting-state fMRI. In generation, if the parameters of the motion are neither the translation shifts of integer voxel counts nor rotation angle about multiples of 90°, voxel values must be interpolated from neighboring voxel values. In order to achieve such transformation without resampling error, theoretically an infinite sinc function should be selected to convolve with interstitial data values in image domain. However, considering the finite extent of MR images as well as related computational burden for such “ideal” interpolation, it is impossible to implement in current computing systems. Therefore, the resampling error always exits to certain degree and affects the accuracy of fMRI images.

It was observed that both the amplitude of SV and the amplitude ratio between SV and BOLD signal were more likely to have larger values in the frontal cortex and occipital gyrus. Several hypotheses may be used to explain this effect. Firstly, these regions are located around the edges of the brain, indicating that high contrast edges may be responsible for this result. Furthermore, as the resampling error could be magnified among neighboring voxels with high contrast, spatial resampling is expected to be an additional source for the larger values around these regions. Taking the change in mean intensity of a voxel as a measure of the amount of resampling, the relationship between the amount of resampling and the magnitude of spurious variance was investigated. It was found that there was no obvious linear relationship exists, but all voxels within these affected regions (which had relatively larger amplitude of SV) showed a relatively larger amount of resampling. Finally, the spatial distribution of the amplitudes of SV (Figure 3) showed that most areas of frontal cortex were more likely to have large values, while parietal lobule were less likely so. It is also worth pointing out that the greatest motion appears in the frontal cortex and the least appears in the parietal cortex, as nodding is the most common head movement pattern. A reasonable explanation would be that there is a difference between translations and rotations in their ability to generate spurious variance, and that the major effect from rotation could account for the similarity of spatial distribution between SV and voxel-specific head motion.

Generally, motion-BOLD relationship has been regarded as either motion-associated neuronal activity and/or motion-induced artifacts (Power et al., 2012; Yan et al., 2013). However, our simulation suggested that processing procedures themselves such as volumetric registration could also contribute confounded signals to signal series. Coupled with the significant correlation between the induced SV and motion, the influence of processing procedures might also be taken account into a comprehensive interpretation of motion-BOLD relationship.

It is worth noting that the SV-motion correlation should be theoretically consistent for simulated minor and abrupt motion data, however, their simulation results were quite different (Figure 5). During the simulation, the only difference between these two data sets was the frame order, but such a frame order difference should not lead to different SV-motion correlation coefficients. According to the data processing flow chart in Figure 1, the frame order should be the only difference between minor and abrupt motion datasets after motion correction, and we inferred that the procedure of polynomial trends regression might turn this frame order difference in time series into different inter-group statistical results in MNI space. This inference was proved to be reasonable, as the motion-SV correlation relationships were basically the same between the minor and abrupt motion data sets if the polynomial trends regression was not included in the process. Since the influence of polynomial trends regression procedure is beyond our focus in this paper, the result is not shown. Specifically, this procedure decreased the intra-group significantly correlated areas; however, it produced potential inter-group difference as a consequence of differences in head motion.

There are a few limitations in our simulation that should be acknowledged. First, it is important to note that motion estimation in human data could be even more complicated than that in the simulation. Regarding this problem, the accuracy of realignment methods could be improved by tracking head motion via a camera or even adjusting the gradients at real time for online motion-correction (Maclaren et al., 2013). Second, as we focused on the influence of spatial resampling, many practical aspects, such as spin history, continuous motion and physiologic signals, were not considered in the current work. To some extent, a complicated and realistic design of simulation is meaningful for better guidance of real-world situations. Our successive work would incorporate some noise elements into data simulation, making the results more representative. Third, many strategies were proposed to eliminate motion-related variance, and their performances were examined by using the change of signal intensity and RSFC correlations (Power et al., 2014; Siegel et al., 2016). It should be emphasized that motion-related signal changes were not effectively reduced by a variety of motion-based regression approaches, even those with very high model orders (Satterthwaite et al., 2013; Power et al., 2014). Therefore, other strategies (e.g. motion censoring, global signal regression) should be evaluated and compared with regression strategies in further simulation studies. Finally, although we focused on delineating the intertwined effects of spatial resampling in resting-state fMRI analysis, the good merits of image realignment still deserve affirmation to achieve meaningful statistical inference.

## Conclusion

The simulation experiments in this paper were designed to demonstrate the influence of spatial resampling on BOLD signal, and four regression approaches were applied to remove SV from the data. As a detectable effect from spatial resampling was found, the interpretation of fMRI results around the edges of brain should be prudent, especially based on amplitude statistics. Moreover, the influence of spatial resampling also suggests an alternative explanation for motion-BOLD relationship, in addition to motion-associated neuronal activity and/or motion-induced artifacts. Furthermore, spurious effects from spatial resampling are substantially reduced but not eliminated by regression approaches. Among them, higher-order regression approaches (Friston 24 and Voxel-specific 12) are more effective than lower-order regression approaches. To sum up, by separating related effects during fMRI analysis, our simulation provides a better understanding of the characteristics of spatial resampling and the interpretation of motion-BOLD relationship.

## Ethics statement

This study was carried out in accordance with the recommendations of the research ethics review board of Hangzhou Normal University. All participants were provided informed written consent and signed it before the MRI scans. No any vulnerable populations were involved.

## Author contributions

LY analyzed the data and wrote the manuscript, HH designed the study and contributed partly to the manuscript, HZ collected the functional MRI data, and JZ provided critical revision for the framework and academic writing of the manuscript.

## Funding

This work has been funded by the National Natural Science Foundation of China No. 81401473, 81201156, and 91632109.

### Conflict of interest statement

The authors declare that the research was conducted in the absence of any commercial or financial relationships that could be construed as a potential conflict of interest.

## Abbreviations

fMRI: functional magnetic resonance imaging
BOLD: blood oxygen level dependent
WM: white matter
CSF: cerebral spinal fluid
ALFF: amplitude of low frequency fluctuation
MNI: Montreal Neurological Institute
FWHM: full-width half-maximum
SD: the standard deviation
SV: spurious variance
TD_vox_: the voxel-wise total displacement
SD_mean_: the mean of SD values within the brain.
