# Supplementary material for: Evaluating the Influence of Spatial Resampling for Motion Correction in Resting-State Functional MRI
Source: Front Neurosci. 2016 Dec 27;10:591. doi: 10.3389/fnins.2016.00591 (PMC5186805; doi:10.3389/fnins.2016.00591)
Supplement: Supplementary file 1 [file Table1.DOCX]

Table S1. The merits of four models were assessed with one-way ANOVA and multiple comparison of Bonferroni’s correction on the mean positive / negative correlation z values of forty-four simulated data in ***minor motion*** type.

| **Minor motion** | (I) Models | (J) Models | Mean difference | SD | Bonferroni |
| --- | --- | --- | --- | --- | --- |
| **Negative correlation**  F(3,172) = 7.47  P = 9.9E-5 | Rigidbody 6 | Derivative 12 | -0.00016 | 0.000633 | 1.000000 |
|  |  | Friston 24 | -0.00224* | 0.000633 | 0.003 |
|  |  | Voxelspecific 12 | -0.00215* | 0.000633 | 0.005 |
|  | Derivative 12 | Friston 24 | -0.002077* | 0.000633 | 0.007500 |
|  |  | Voxelspecific 12 | -0.001989* | 0.000633 | 0.011791 |
|  | Friston 24 | Voxelspecific 12 | 0.000087 | 0.000633 | 1.000000 |
| **Positive Correlation**  F(3,172) = 7.36  P = 1.1E-4 | Rigidbody 6 | Derivative 12 | 0.000133 | 0.000510 | 1.000000 |
|  |  | Friston 24 | 0.001802* | 0.000510 | 0.003183 |
|  |  | Voxelspecific 12 | 0.001715* | 0.000510 | 0.005746 |
|  | Derivative 12 | Friston 24 | 0.001670* | 0.000510 | 0.007749 |
|  |  | Voxelspecific 12 | 0.001583* | 0.000510 | 0.013523 |
|  | Friston 24 | Voxelspecific 12 | -0.000087 | 0.000510 | 1.000000 |
