# Supplementary material for: Evaluating the Influence of Spatial Resampling for Motion Correction in Resting-State Functional MRI
Source: Front Neurosci. 2016 Dec 27;10:591. doi: 10.3389/fnins.2016.00591 (PMC5186805; doi:10.3389/fnins.2016.00591)
Supplement: Supplementary file 2 [file Table2.DOCX]

Table S2. The merits of four models were assessed with one-way ANOVA and multiple comparison of Bonferroni’s correction on the mean positive / negative correlation z values of forty-four simulated data in ***abrupt motion*** type.

| **Abrupt motion** | (I) Models | (J) Models | Mean difference | SD | Bonferroni |
| --- | --- | --- | --- | --- | --- |
| **Negative Correlation**  F(3,172) = 7.717  P = 7.2E-5 | Rigidbody 6 | Derivative 12 | -0.000057 | 0.000681 | 1.000000 |
|  |  | Friston 24 | -0.002447* | 0.000681 | 0.002535 |
|  |  | Voxelspecific 12 | -0.002230* | 0.000681 | 0.007624 |
|  | Derivative 12 | Friston 24 | -0.002390* | 0.000681 | 0.003408 |
|  |  | Voxelspecific 12 | -0.002173* | 0.000681 | 0.010043 |
|  | Friston 24 | Voxelspecific 12 | 0.000217 | 0.000681 | 1.000000 |
| **Positive Correlation**  F(3,172) = 6.751  P = 2.5E-4 | Rigidbody 6 | Derivative 12 | 0.000047 | 0.000607 | 1.000000 |
|  |  | Friston 24 | 0.002134* | 0.000607 | 0.003403 |
|  |  | Voxelspecific 12 | 0.001738* | 0.000607 | 0.028427 |
|  | Derivative 12 | Friston 24 | 0.002087* | 0.000607 | 0.004459 |
|  |  | Voxelspecific 12 | 0.001691* | 0.000607 | 0.035827 |
|  | Friston 24 | Voxelspecific 12 | -0.000395 | 0.000607 | 1.000000 |
