# Supplementary material for: Evaluating the Influence of Spatial Resampling for Motion Correction in Resting-State Functional MRI
Source: Front Neurosci. 2016 Dec 27;10:591. doi: 10.3389/fnins.2016.00591 (PMC5186805; doi:10.3389/fnins.2016.00591)
Supplement: Supplementary file 3 [file Table3.DOCX]

Table S3. The merits of four models were assessed with one-way ANOVA and multiple comparison of Bonferroni’s correction on the mean positive / negative correlation z values of forty-four simulated data in ***big-spike motion*** type.

| **Big-spike motion** | (I) Models | (J) Models | Mean difference | SD | Bonferroni |
| --- | --- | --- | --- | --- | --- |
| Negative Correlation  F(3,172) = 3.308  P = 0.022 | Rigidbody 6 | Derivative 12 | -0.000071 | 0.000532 | 1.000000 |
|  |  | Friston 24 | -0.001408 | 0.000532 | 0.053616 |
|  |  | Voxelspecific 12 | -0.000937 | 0.000532 | 0.481353 |
|  | Derivative 12 | Friston 24 | -0.001337 | 0.000532 | 0.077536 |
|  |  | Voxelspecific 12 | -0.000866 | 0.000532 | 0.633063 |
|  | Friston 24 | Voxelspecific 12 | 0.000471 | 0.000532 | 1.000000 |
| Positive Correlation  F(3,172) = 3.56  P = 0.016 | Rigidbody 6 | Derivative 12 | 0.000081 | 0.000464 | 1.000000 |
|  |  | Friston 24 | 0.001305* | 0.000464 | 0.033097 |
|  |  | Voxelspecific 12 | 0.000791 | 0.000464 | 0.542766 |
|  | Derivative 12 | Friston 24 | 0.001224 | 0.000464 | 0.054965 |
|  |  | Voxelspecific 12 | 0.000709 | 0.000464 | 0.770694 |
|  | Friston 24 | Voxelspecific 12 | -0.000515 | 0.000464 | 1.000000 |
