# Supplementary material for: Evaluating the Influence of Spatial Resampling for Motion Correction in Resting-State Functional MRI
Source: Front Neurosci. 2016 Dec 27;10:591. doi: 10.3389/fnins.2016.00591 (PMC5186805; doi:10.3389/fnins.2016.00591)
Supplement: Supplementary file 4 [file Table4.DOCX]

Table S4. The merits of four models were assessed with one-way ANOVA and multiple comparison of Bonferroni’s correction on the SD_mean_ values of forty-four simulated data for all types of motion.

| **SD_mean_** | (I) Models | (J) Models | Mean difference | SD | Bonferroni |
| --- | --- | --- | --- | --- | --- |
| **Minor motion**  F(3,172) = 5.615  P = 0.0011 | Rigidbody 6 | Derivative 12 | 0.016949 | 0.049192 | 1.000000 |
|  |  | Friston 24 | 0.158802* | 0.049192 | 0.008952 |
|  |  | Voxelspecific 12 | 0.141637* | 0.049192 | 0.026955 |
|  | Derivative 12 | Friston 24 | 0.141853* | 0.049192 | 0.026600 |
|  |  | Voxelspecific 12 | 0.124688 | 0.049192 | 0.072874 |
|  | Friston 24 | Voxelspecific 12 | -0.017165 | 0.049192 | 1.000000 |
| **Abrupt motion**  F(3,172) = 8.375  P = 3.1E-5 | Rigidbody 6 | Derivative 12 | 0.009606 | 0.054341 | 1.000000 |
|  |  | Friston 24 | 0.207388* | 0.054341 | 0.001132 |
|  |  | Voxelspecific 12 | 0.185996* | 0.054341 | 0.004648 |
|  | Derivative 12 | Friston 24 | 0.197782* | 0.054341 | 0.002165 |
|  |  | Voxelspecific 12 | 0.176390* | 0.054341 | 0.008442 |
|  | Friston 24 | Voxelspecific 12 | -0.021392 | 0.054341 | 1.000000 |
| **Big-spike motion**  F(3,172) = 20.02  P = 3.6E-11 | Rigidbody 6 | Derivative 12 | 0.074748 | 0.068899 | 1.000000 |
|  |  | Friston 24 | 0.449059* | 0.068899 | 4.57E-9 |
|  |  | Voxelspecific 12 | 0.363441* | 0.068899 | 0.000002 |
|  | Derivative 12 | Friston 24 | 0.374311* | 0.068899 | 0.000001 |
|  |  | Voxelspecific 12 | 0.288694* | 0.068899 | 0.000267 |
|  | Friston 24 | Voxelspecific 12 | -0.085618 | 0.068899 | 1.000000 |
